# Biodistribution and Tumor Uptake of ^67^Ga-Nimotuzumab in a Malignant Pleural Mesothelioma Xenograft

**DOI:** 10.3390/molecules23123138

**Published:** 2018-11-29

**Authors:** Vanessa Izquierdo-Sánchez, Saé Muñiz-Hernández, Héctor Vázquez-Becerra, Judith Pacheco-Yepez, Mario E. Romero-Piña, Oscar Arrieta, Luis Alberto Medina

**Affiliations:** 1 Sección de Estudios de Posgrado e Investigación, Escuela Superior de Medicina, Instituto Politécnico Nacional, Ciudad de México 11340, Mexico; van.izq.san@gmail.com (V.I.-S.); payejuca@prodigy.net.mx (J.P.-Y.); 2 Unidad de Investigación Biomédica en Cáncer, INCan/UNAM, Instituto Nacional de Cancerología (INCan), Ciudad de México 14080, Mexico; hekator@gmail.com (H.V.-B.); esau1708@gmail.com (M.E.R.-P.); 3 Laboratorio de Oncología Experimental, Subdirección de Investigación Básica, Instituto Nacional de Cancerología, Ciudad de México 14080, Mexico; sayide@hotmail.com (S.M.-H.); ogarrieta@gmail.com (O.A.); 4 Instituto de Física, Universidad Nacional Autónoma de México (UNAM), Ciudad de México 04510, Mexico; 5 Unidad de Oncología Torácica, Instituto Nacional de Cancerología, Ciudad de México 14080, Mexico

**Keywords:** nimotuzumab, malignant pleural mesothelioma, radioimmunoconjugates, biomarkers, molecular imaging

## Abstract

Malignant pleural mesothelioma (MPM) is the most common tumor of the pulmonary pleura. It is a rare and aggressive malignancy, generally associated with continuous occupational exposure to asbestos. Only a multimodal-approach to treatment, based on surgical resection, chemotherapy and/or radiation, has shown some benefits. However, the survival rate remains low. Nimotuzumab (h-R3), an anti-EGFR (epidermal growth factor receptor) humanized antibody, is proposed as a promising agent for the treatment of MPM. The aim of this research was to implement a procedure for nimotuzumab radiolabeling to evaluate its biodistribution and affinity for EGF (epidermal growth factor) receptors present in a mesothelioma xenograft. Nimotuzumab was radiolabeled with ^67^Ga; radiolabel efficiency, radiochemical purity, serum stability, and biodistribution were evaluated. Biodistribution and tumor uptake imaging studies by microSPECT/CT in mesothelioma xenografts revealed constant nimotuzumab uptake at the tumor site during the first 48 h after drug administration. In vivo studies using MPM xenografts showed a significant uptake of this radioimmunoconjugate, which illustrates its potential as a biomarker that could promote its theranostic use in patients with MPM.

## 1. Introduction

Malignant pleural mesothelioma (MPM) is the most common tumor of the pulmonary pleura. It is a rare and aggressive malignancy, usually associated with continuous occupational exposure to asbestos, others etiological factors including iron, the simian virus 40 (SV40), and radiation [1,2,3,4]. Its main characteristic is an aggressive progression in almost all cases and a subsequent poor prognosis [1]. This tumor shows low chemo and radio-sensitivity [4]. Only a multimodal-approach based on surgical resection and a combination of chemotherapy and radiation has shown some benefits. However, the survival rate remains low. The best response in patients has been found when combinations of chemotherapeutics are prescribed, mainly cisplatin and pemetrexed [5,6,7,8,9,10]. 

Alternative treatments for MPM have been reported in preclinical and early clinical studies. Some of these studies include the use of tyrosine kinase inhibitors, anti-mesothelin antibodies, immunotoxin therapies, antagonist antibodies of immune checkpoints, genetically modified T cells, viruses, and liposomes [11,12,13,14,15]. Lately, vascular endothelial growth factor receptor (VEGFR) and the epidermal growth factor receptor (EGFR) have been reported as potential targets for anticancer therapies [16,17,18]; its overexpression in mesothelioma has been also reported [14,19,20,21]. Based on this, many therapeutic agents, including the monoclonal antibodies bevacizumab (anti-VEGF), panitumumab, and cetuximab (anti-EGFR), have been proposed for the treatment of MPM, showing some therapeutic response [4,22,23]. Recently, nimotuzumab (h-R3), an anti-EGFR humanized antibody, has shown promising results in the treatment of head and neck, glioma, lung, pancreatic, and gastric cancers [24,25]. This agent has shown a good toxicity profile with a low incidence of adverse events (rash grade 1/2) [26,27,28]. These results suggest exploring the use of nimotuzumab in the treatment of MPM. However, a preclinical evaluation is necessary before its clinical use.

Molecular imaging techniques are non-invasive assessments used for the in vivo characterization and measurement of biological processes at the cellular and molecular level. They could be suitable to help understand the biodistribution and uptake of radiolabeled nimotuzumab by tumoral cells in MPM, overexpressing the targeted receptor. In vivo studies require gamma-emitter radionuclides with a physical half-life (t_1/2_) that matches the in vivo kinetics of the antibody [29,30,31]. In patients with advanced solid tumors, the biological half-life (t_1/2_) of nimotuzumab is 34 to 75 h, depending on the injected dose (100 to 400 mg) [27]; this value suggests the use of gamma-emitter radionuclides with a physical half-life in the same range. Gallium-67 (^67^Ga) is a potential radionuclide that can be used to investigate the biodistribution of nimotuzumab, since its physical half-life is 78.25 h. Its gamma emissions (γ-ray energy = 93 keV (39%), 184 keV (21%), 300 kev (17%), and 393 keV (5%)) allow obtaining images with a gamma camera, a SPECT camera or with SPECT/CT hybrid systems [32].

The aim of this research was to implement a procedure for nimotuzumab radiolabeling with ^67^Ga, and evaluate its biodistribution and affinity for EGFR receptors present in a mesothelioma xenograft. The final goal is to explore its potential as a biomarker that could promote its use as a theranostic agent in patients with MPM.

## 2. Results

### 2.1. Radiolabeling

The radiolabeling efficiency of the ^67^Ga-nimotuzumab radioimmunoconjugate (RIC) was 45 ± 4% (mean ± S.D., *n* = 5). The radiochemical purity was 92.4 ± 1.4% (*n* = 5). The in vitro stability profile in human serum at 37 °C after 72 h was about 98 ± 2%.

### 2.2. Antibody Integrity

**Figure 1:**
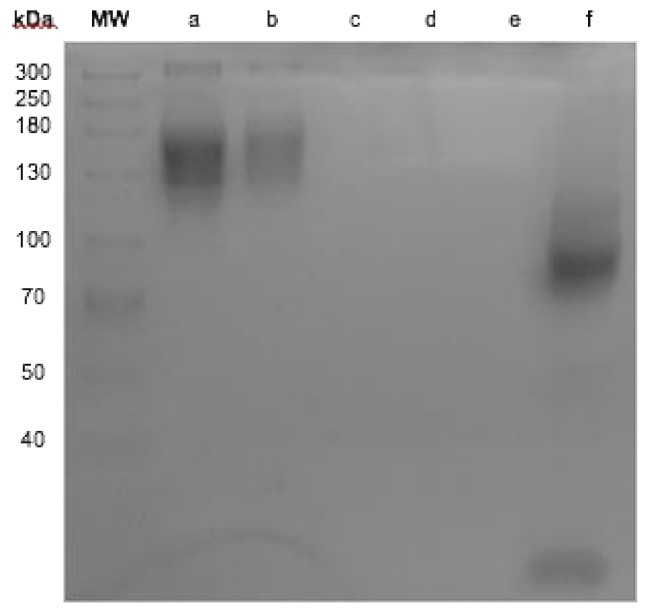
SDS-PAGE. Molecular weight size marker, (**a**) Nimotuzumab, (**b**) conjugate diethylenetriaminepentaacetic acid (DTPA)-nimotuzumab, (**c**) residual DTPA after purification, (**d**) DTPA, (**f**) nimotuzumab semi-reduction conditions.

It was observed that the bifunctional chelator, diethylenetriaminepentaacetic acid (DTPA), attached to nimotuzumab does not have any effect on its integrity and no antibody fragments were found as result of the conjugation process (Figure 1, column b and c). When nimotuzumab was processed in reducing conditions with β-mercaptoethanol, a fragment of 90 kDa and another with 15 kDa were seen during the SDS-PAGE process (Figure 1, column f).

### 2.3. Cell Binding Assay

Experiments were performed to test the ability of the RIC to bind cell lines that overexpress EGFR. The binding efficiency of ^67^Ga-Nimotuzumab in MRC-5 (cells with basal levels of EGFR), A431 (cells with EGFR-overexpression), and MSTO-211H (malignant pleural mesothelioma) cells was 6.9 ± 0.3%, 34.5 ± 1.9%, and 21.6 ± 1%, respectively. The non-specific binding to cells was lower than 7%. 

### 2.4. Biodistribution and Tumor Uptake in Mesothelioma Xenografts

**Figure 2:**
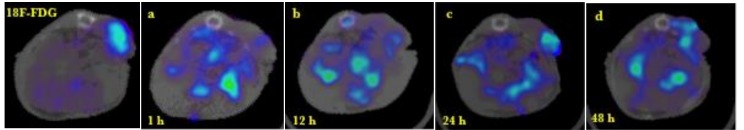
Left: The ^18^FDG PET/CT image denotes the metabolic activity of a mesothelioma xenograft. Images **a**–**d**: ^67^Ga-nimotuzumab SPECT/CT shows nimotuzumab uptake in the tumor at 1, 12, 24, and 48 h post-injection. All images show transversal projections based on the tumor position of the same animal at different times.

Representative images of the tumor and normal tissue biodistribution of ^67^Ga-nimotuzumab are shown in Figure 2. The ^18^FDG PET/CT molecular image (left) depicts tumor metabolic activity while the SPECT/CT images (a–d) show ^67^Ga-nimotuzumab uptake in tumor and bowel tissue at different times post-injection. Contrary to ^18^FDG, the RIC uptake in tumor tissue was not homogeneous at any time. All images correspond to the same mouse, were acquired at different times, and are representative of the whole group (*n* = 3). The PET/CT image shows increased ^18^FDG uptake in the tumor tissue as compared to liver tissue. The liver was the standard reference organ for ^18^FDG metabolism, indicating tumor metabolic activity during the experiments. 

**Figure 3:**
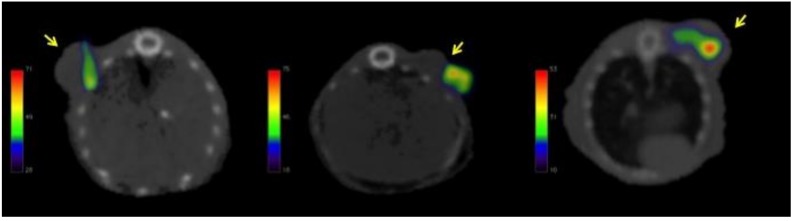
Representative transversal projection of each animal illustrating the tumor uptake of ^67^Ga-nimotuzumab at 24 h post-injection. Arrows point to tumor location.

Figure 3 illustrates tumor uptake of ^67^Ga-nimotuzumab at 24 h post-injection. A representative transversal projection of each animal is shown. Due to the different position of the tumor in each animal, the background distribution of the radioimmunoconjugate was removed from the images to highlight tumor uptake.

Table 1 shows the quantitative analysis of the SPECT/CT images at different times. The percentage of nimotuzumab uptake in the tumor tissue was constant over 48 h; no statistical differences were observed between time-points, but it seems there was a maximal accumulation at 24 h. The uptake ratio between the tumor and liver tissues was greater than five at all times.

**Figure 4:**
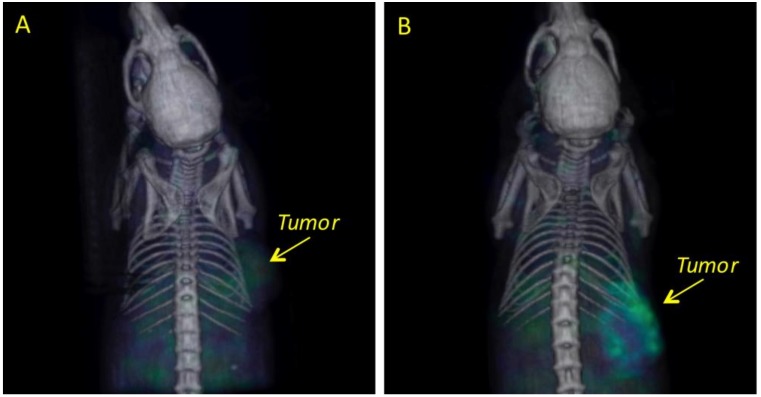
Representative SPECT/CT 3D-images showing the differences in biodistribution and uptake in mesothelioma xenografts at 24 h post-injection for: (**A**) ^67^Ga (control group) and (**B**) ^67^Ga-nimotuzumab.

The biodistribution of ^67^GaCl_3_ in the liver and bowel is shown in Figure 4A; low accumulation in the tumor was observed. ^67^Ga-nimotuzumab uptake was observed in the tumor, with minimal accumulation in the gastrointestinal system (Figure 4B).

**Figure 5:**
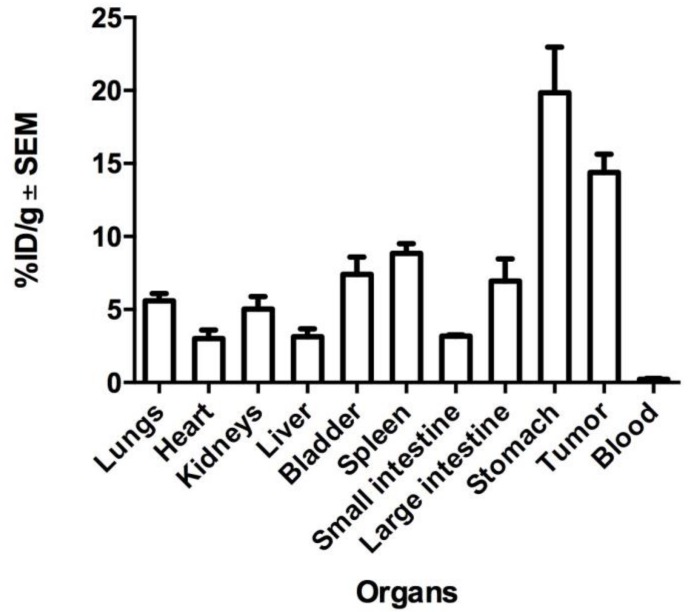
Biodistribution profile of ^67^Ga-nimotuzumab in the tumor and organs of interest at 48 h. Values represent the mean ±SEM (*n* = 3).

An organ biodistribution profile of nimotuzumab (%ID/g) after 48 h is shown in Figure 5. Data confirm uptake in organs associated with the metabolism and clearance of monoclonal antibodies (mAbs), namely liver (3%), spleen (9%), kidneys (5%), but also accumulation in the small intestine (3%), large intestine (7%), stomach (20%), bladder (7%), heart (3%), and lungs (6%). High uptake of ^67^Ga-nimotuzumab (14%) was measured in the tumor.

## 3. Discussion

Therapeutic options for a single treatment modality for MPM, such as chemotherapy, radiation, surgery, immunotherapy, or palliative procedures, have not shown good results, with survival rates from 6–20 months from diagnosis [23,33,34,35]. More promising results have been obtained using a combination of chemo- and immunotherapy [6,7,9,14]. Nimotuzumab may serve as a novel option to treat MPM, but complete knowledge of its biodistribution in tumor tissue and organs of interest is required before it can be used in immunotherapy, alone, or in combination with standard chemotherapy.

In the present study, a radiolabeling procedure for nimotuzumab with ^67^Ga was implemented to evaluate its biodistribution and uptake in MPM xenografts by molecular imaging techniques. The use of radionuclides with long t_1/2_ helps to track the antibody uptake for a longer time (more than 24 h). The physical and radiological characteristics of ^67^Ga (t_1/2_ = 78.25 h and main gamma emissions of 93, 184, and 300 keV) [32], and its availability and low cost make this radionuclide a potential candidate to be used in radiolabeling and imaging procedures with mAbs. ^111^In is another radionuclide with radiological characteristics that make it more suitable for molecular imaging studies (t_1/2_ = 2.8 days, and gamma emission of 173 and 247 keV); however, its cost and availability could limit its use.

The efficient radiolabeling of mAbs depends on some critical points, such as heating temperature, molar proportion mAb, chelating agent, incubation time, pH, and reaction time [30]. DTPA easily attaches to the antibody without special temperature conditions and it can be handled at room temperature, facilitating its use. Although other chelators, for example DOTA (1,4,7,10-Tetraazacyclododecane-N,N′,N″,N‴-tetraacetic acid) and NOTA (1,4,7-triazacyclononane-1,4,7-trisacetic acid), have been used to radiolabel monoclonal antibodies, the availability and low cost of DTPA motivated its use in the present work [30,31,32].

It has been reported that the use of a molar ratio 1:10 (antibody/DTPA) results in a chelation of about two groups of DTPA per antibody, other groups have reported between 0.9 to 5 DTPAs per antibody molecule [36,37]; this difference can be attributed to the number of lysines in the structure and those available to react. Nimotuzumab possesses nine lysines [38]; however, not all of them can react with DTPA because the tertiary structure is a three-dimensional folding that results from interactions between elements such as disulfide bridges, hydrophobic effects, charge–charge interactions, H-bonding, and van der Waal interactions. Thus, this configuration can hide some lysines and limit their reaction [39].

The conjugation time did not affect chelation, we did not observe any differences in the chelation efficiency after 0.5, 1 and 2 h. The radiolabeling efficiency (around 50%) was sufficient to perform imaging studies. The radiochemical purity was 92.4 ± 1.4% and the stability in serum after 72 h was about 98%, even when ethylenediaminetetraacetic acid (EDTA) was used as a competitor. Although some reports have mentioned that the in vitro dissociation of the radionuclide by trans-complexation and transferring could reduce the stability of the radioimmunoconjugate [30,40,41], radiolabeling stability was maintained in our study. SDS-PAGE was performed to demonstrate that the chelation process did not affect the integrity of nimotuzumab (Figure 1). The results showed that nimotuzumab preserved its integrity throughout the process, which included a conjugation buffer with basic pH and acid pH adjustment for radiolabeling. 

In the cell binding assay, we used the A431 cell line that overexpresses EGFR on their surface (2 × 10^6^ receptors by cell) [42], we found an elevated percentage of RIC binding efficiency in the cell line MSTO-211H (biphasic mesothelioma cells), with less expression of EGFR (34 vs. 21%). These results are similar to a study in which nimotuzumab was radiolabeled with ^131^I, where the in vitro efficiency binding in A431 cells was 28 ± 0.7% [43]. Our results suggest that the cell-binding of ^67^Ga-nimotuzumab is not compromised by the chelation process, and the radiolabeled antibody maintains affinity and specificity for the EGFR expressed in MPM cells.

Radiotracers are specific, radiolabeled molecules (probes) that resemble the in vivo behavior of the molecule (e.g., a monoclonal antibody) to provide information about a specific biological process. A common problem associated with a radiotracer is altered biodistribution that can impact on safety, image interpretation, and diagnostic accuracy, which can compromise the utility of molecular imaging studies [44]. Imaging studies of ^67^Ga-nimotuzumab are important to provide information on the whole-body distribution of nimotuzumab, to elucidate its target expression in MPM, uptake in the tumor, tumor saturation, and heterogeneity for these parameters within the tumor.

A main limitation of our work was the reduced number of animals used (*n* = 3) in the experiment; however, the results are still valuable and representative of the biodistribution process of nimotuzumab. The accumulation of ^67^Ga-nimotuzumab in selected organs 48 h after intravenous administration (Figure 5), show similar values to those reported in other studies of nimotuzumab radiolabeled with ^177^Lu, ^131^I, ^111^In [41,43,45,46]. The uptake in the liver, kidney, and spleen indicate a clearance process that is consistent with other reports [47,48]. The tumor-to-liver uptake ratio (5:1), estimated in images at different times (Table 1), indicates a specific targeting of nimotuzumab for mesothelioma xenograft, in comparison with the accumulation in normal (healthy) reference tissue (liver). The accumulation of ^67^Ga-nimotuzumab in MPM xenografts was constant during the whole study (Figure 2 and Figure 3), this result is important and should be considered for further studies.

The uptake of the free radionuclide (^67^GaCl_3_) in mesothelioma xenografts was different when compared with ^67^Ga-nimotuzumab; a mild tumor uptake of ^67^GaCl_3_ was observed at 24 h post-injection (Figure 4). Because ^67^GaCl_3_ does not have a specific target in the body, its small size facilitates its clearance through the kidneys, which is the main route of elimination for small water-soluble molecules with low molecular weight [47]. Konikowski et al. [49] have reported a 30% (% dose) cumulative urinary excretion in mice, two hours after a single i.v. injection of ^67^GaCl_3_; they also report constant values (about 5% ID/g) of kidney concentration of the free radionuclide during the following two hours after injection (no values were reported after 2 h). In contrast, it has been reported that nimotuzumab is not directly excreted in the urine or metabolized by the microsomal hepatic system, maybe as result of its high molecular weight (150,000 kDa) [48,50,51]. The elimination route of antibodies has not been totally elucidated but several mechanisms have been proposed; for example, proteolysis in the liver and reticuloendothelial systems could result in the fractionation of the antibody into peptides and amino acids, which can be reused for the synthesis of new proteins or excreted in urine. Furthermore, target elimination and nonspecific endocytosis have also been proposed [48,50,51]. 

Antibody biodistribution mainly depends on the molecular weight and the affinity for specific receptors, however, uptake in tumor cells could also be limited by the tumor physiology, vasculature, and irregular structure [47]. In our experiment, the inhomogeneous tumor uptake of ^67^Ga-nimotuzumab was observed in the mesothelioma xenografts (Figure 3 and Figure 4). In a more detailed experiment, which should include a histopathological study, ^67^Ga-nimotuzumab could be used to elucidate whether MPM tumors with limited vasculature and a large amount of fibrous tissue would reduce nimotuzumab penetration. Imaging studies with ^67^Ga-nimotuzumab could help to estimate treatment response in tumors with these characteristics.

Radiolabeled mAbs have gained relevance for their safe, good quality imaging in nuclear medicine procedures, and availability for diagnostics and therapy [46,52]. The use of beta-particle emitter radionuclides (^177^Lu, ^131^I, ^188^Re) in the radiolabeling of nimotuzumab could promote its use as a radioimmunotherapeutic agent for the treatment of malignant pleural mesothelioma.

## 4. Materials and Methods

### 4.1. Reagents

Nimotuzumab was acquired from Pisa Pharmaceutics (Mexico City), the chelating diethylenetriaminepentaacetic acid (DTPA) was obtained from Sigma-Aldrich (St. Louis, MO, USA). Dulbecco´s modified Eagle’s/F12 medium (DMEM/F12), F12 medium, fetal bovine serum (FBS), ethylenediaminetetraacetic acid (EDTA), and sodium dodecyl sulfate (SDS) were obtained from Gibco (Waltham, MA, USA). Amicon columns 30 kDa was from Merck-Millipore. High-quality water employed to prepare solutions was obtained through a Milli-Q Reagent Water System (Continental Water Systems; El Paso, TX, USA). All other chemicals were purchased from Sigma-Aldrich (St. Louis, MO, USA) and Promega (Madison, WI, USA). The radionuclide (^67^GaCl_3_) was purchased from a local radiopharmacy (MYMSA, Ciudad de México, Mexico).

### 4.2. Animals

Male athymic nu-/nu- mice, between 5–6 weeks of age, were obtained from Unidad de Producción y Experimentación de Animales de Laboratorio (UPEAL-CINVESTAV, Ciudad de México, México). Animals were kept in a pathogen-free environment in a microisolator and fed with autoclaved food and water ad libitum. The procedures for care and use of the animals were approved by a local institutional Ethics Committee (017/027/IBI) (CEI/1147/17) and all applicable institutional and governmental regulations were followed, in accordance with the Mexican Federal Regulations for Animal Production, Care and Experimentation (NOM-062-ZOO-1999, Ministry of Agriculture; Mexico City, Mexico). The guidelines from the Guide for the Care and Use of Laboratory Animals of the National Institute of Health (NIH, USA) were also followed. All efforts were made to minimize animal suffering and to reduce the number of animals used.

### 4.3. Antibody Conjugation with DTPA

Conjugation was performed using the methods reported previously [36,37,40]. Briefly, the suspension buffer of nimotuzumab was exchanged for sodium bicarbonate (0.1 M, pH 9.0) using an Amicon Ultra-0.5 centrifugal filter unit with Ultracel-30 kDa. Nimotuzumab (400 μg) in sodium bicarbonate was conjugated at a 1:25 molar ratio using a DTPA solution in dimetylsulfoxide (DMSO) (5 mg/mL). DTPA was added to nimotuzumab and gently mixed in a rocking shaker at room temperature for 1 h. The excess chelator was twice removed by centrifugation (5 min, 7000× *g*) using an Amicon Ultra-0.5 centrifugal filter unit in ammonium acetate buffer (0.1 M, pH 5.0).

### 4.4. Nimotuzumab Integrity Test

After conjugation with DTPA, samples were collected and mixed with a loading buffer (glycerol, sodium dodecyl sulfate (SDS) in Tris-HCl 0.5 M pH 6.8) in non-reducing or reducing conditions (β-mercaptoethanol). Electrophoresis was performed on SDS-polyacrylamide (SDS-PAGE) using 7.5% acrylamide/bis-acrylamide gel in a Biorad Electrophoretical System (Mini-Protean Tetra Vertical Electrophoresis Cell, Hercules, CA, USA). We used nimotuzumab in semi-reduction conditions as a positive denaturalization.

### 4.5. Radiolabeling

The ^67^GaCl_3_ was adjusted to pH 5 with ammonium acetate (2 M, pH 5). The radionuclide (185 MBq) was added and gently mixed to 400 μg of nimotuzumab-DTPA immunoconjugate (IC) and incubated in a dry bath during 1 h at 37 °C. The radioimmunoconjugate (RIC) ^67^Ga-DTPA-nimotuzumab (^67^Ga-nimotuzumab) was purified by centrifugation to eliminate excess ^67^Ga-free, and the buffer was changed to saline solution 0.9%. The radiolabeling procedure was performed in a Biohazard Laminar Air Flow Hood (Nuaire, Plymouth, MN, USA); glassware, materials, and solutions for the labeling procedure were sterile, and pyrogen and metal free.

The radiochemical purity of ^67^Ga-nimotuzumab was evaluated by instant thin layer chromatography on silica-impregnated glass fiber sheets (ITLC-SG) (General Electric, Santa Clara, CA, USA) using a mobile phase of ammonium acetate (0.1 M, pH 5) buffer containing EDTA 1 μM. Briefly, a drop of RIC (1 μL) was added on the ITLC-SG paper, it was then air-dried and cut in half. The radioactivity in each section was measured using a well-type gamma counter equipped with a NaI crystal (Ludlum 2200, Sweetwater; TX, USA). Free ^67^Ga moved to the front while ^67^Ga-DTPA-nimotuzumab remained at the starting position.

To determine the radiolabeling stability, 50 µL of RIC was added to 0.45 mL of human serum and incubated in a dry bath at 37 °C for 72 h. Samples were collected at 1, 2, 3, 12, 24, 48, and 72 h, and quantified by ITLC-SG as described above.

### 4.6. In Vitro Binding Nimotuzumab Assay

The cell binding of ^67^Ga-nimotuzumab was evaluated in human epidermoid carcinoma cells A431 (CRL-1555, ATCC, USA) (positive control), lung cells MRC-5 (CCL-171, ATCC, USA) (normal expression of EGFR), and biphasic mesothelioma cells MSTO-211H (CRL-2081, ATCC, USA). The biphasic histologic type overexpresses EGFR and is present in aggressive tumors with poor treatment response in patients [1,33]. Cells were kept in a culture medium, according to ATCC recommendations, supplemented with 8% FBS, and incubated at 37 °C in a 5% CO_2_ atmosphere. At 80% confluence, cells were washed (PBS 1X), trypsinized, and counted in a Neubauer chamber. The cell suspension in PBS (2 × 10^5^ cells/tube) was incubated at 37 °C for 1 h with 4 µg of RIC (0.37 MBq), or the same activity of ^67^Ga as non-specific union. Then, samples were centrifuged (5 min, 70× *g*) and the radioactivity at the cell pellet and supernatant was measured in the gamma counter. The specific binding was calculated as the quotient of cell-bound radioactivity (pellet) to total radioactivity (pellet plus supernatant). This assay was realized in triplicate.

### 4.7. Mesothelioma Xenografts

The mice nu/nu (*n* = 3) were subcutaneously (s.c.) inoculated in the dorsal right flank with 3 × 10^6^ of MSTO-211H cells. Cells were suspended in a 1:1 molar ratio of F12 with matrigel (Basement Membrane Matrix), phenol-red free, and LDEV-free (Corning, New York, NY, USA). Tumor size was measured weekly with a caliper; volume was determined using the equation V = π/6 x (large diameter x [short diameter]^2^). Animals were used for biodistribution and molecular imaging studies when tumors were greater than 50 mm^3^. Because tumor growth from MSTO-211H cells is very slow, at least 10 weeks were necessary to reach that volume.

### 4.8. Biodistribution and Molecular Imaging Studies

Tumor metabolic activity was evaluated by ^18^F-fluorodeoxiglucose (^18^FDG) uptake imaging by PET/CT with a micro PET/SPECT/CT scanner (Albira, Bruker, Spain). For imaging, the animals were anesthetized using isoflurane (1–2% in 100% oxygen) and intravenously injected with ^18^F-FDG (5.5 MBq) in the caudal vein. The PET/CT scan was performed 40 minutes after injection and data were reconstructed with the OSEM reconstruction algorithm (3 iterations, Albira Suite Software). Animals with an active tumor were used two days later to evaluate ^67^Ga-nimotuzumab tumor uptake and biodistribution by SPECT/CT imaging. Animals were also anesthetized with isoflurane and intravenously injected via the caudal vein with 7.4 MBq (50 μL, 50 μg) of ^67^Ga-nimotuzumab. Images were acquired at 1, 12, 24, and 48 h post-injection. SPECT scans were taken with 30 projections per detector (60 projections total) and 30–45 s/projection. CT scans were performed with 400 projections (tube voltage 35 kV and 0.4 mA). After imaging, the mice were euthanized by cervical dislocation and dissected, organs of interest and the tumor were collected, rinsed of residual blood, and weighed; radioactivity was quantified using the well-type gamma counter (Ludlum 2200, TX, USA). Tissue activity was calculated as a three animal average of the percent of injected dose, per gram of tissue (%ID/g).

### 4.9. Statistical Analysis

Values are reported as mean values ±SEM. When necessary, statistical analysis was performed using one-way analysis of variance (ANOVA) on SPSS Base 20.0 software (SPSS Inc., Chicago, IL, USA). Significance was determined at *p* < 0.05. 

## 5. Conclusions

This work has presented a standardized methodology for the radiolabeling of nimotuzumab with ^67^Ga, without significant loss of immunoreactivity. The results of the biodistribution and specific uptake of ^67^Ga-nimotuzumab in mesothelioma xenografts are valuable and will motivate further imaging studies to explore its potential use as a theranostic biomarker in clinical studies of patients with malignant pleural mesothelioma.

## Figures and Tables

**Table 1:** Nimotuzumab uptake in tumor tissue and uptake ratio between tumor and liver tissues.

Time (h)	% Tumor	Tumor/Liver
1	1.52 ± 0.15	6.61 ± 0.42
12	1.44 ± 0.23	6.21 ± 2.26
24	1.82 ± 0.18	8.46 ± 2.48
48	1.47 ± 0.31	5.71 ± 1.95

Values represent the average ±SEM (*n* = 3).
